# Bisphenol a downregulates GLUT4 expression by activating aryl hydrocarbon receptor to exacerbate polycystic ovary syndrome

**DOI:** 10.1186/s12964-023-01410-y

**Published:** 2024-01-10

**Authors:** Jing Shi, Kai-Lun Hu, Xiao-Xue Li, Yi-Meng Ge, Xiao-Jun Yu, Jie Zhao

**Affiliations:** 1 Center for Reproductive Medicine, Department of Obstetrics and Gynecology, Peking University Third Hospital, 49 North Garden Rd, Haidian District, Beijing, 100191 China; 2 Department of Pharmacy, Peking University Third Hospital, Beijing, China; 3 National Clinical Research Center for Obstetrics and Gynecology, Beijing, 100191 China; 4 Key Laboratory of Assisted Reproduction (Peking University), Ministry of Education, Beijing, 100191 China; 5 Beijing Key Laboratory of Reproductive Endocrinology and Assisted Reproductive Technology, Beijing, 100191 China; 6 Faculty of Life Science and Technology, Kunming University of Science and Technology, Kunming, China

**Keywords:** Bisphenol a, Polycystic ovary syndrome, Glucose transporter 4, Aryl hydrocarbon receptor, Ovarian granulosa cells

## Abstract

**Background:**

Bisphenol A (BPA) levels are high in women with polycystic ovary syndrome (PCOS). The mechanism by which BPA induces abnormal glucose metabolism in PCOS patients is largely unknown.

**Methods:**

Serum and urine samples were collected from women with and without PCOS (control) at the reproductive medicine center with informed consent. Non-PCOS patients who received in vitro fertilization were recruited for collection of ovarian follicular fluid and granular cells. Wild-type C57BL/6 and *AhR* ^*−/−*^ mice were used to verify the effects of BPA on PCOS. Real-time PCR, western blotting, and ELISA were conducted to analyze the function of BPA. Chip-qPCR verified the role of AhR in *GLUT4* transcription. Flow cytometry was performed to determine glucose uptake.

**Results:**

A positive correlation was observed between BPA concentration and serum BPA levels in PCOS patients. BPA aggravated the changes in PCOS with abnormal glucose metabolism, impaired fertility, and increased body fat. Mechanistically, we showed that BPA activated AhR and led to decreased glucose transport via GLUT4 downregulation in ovarian granular cells. Therefore, the use of inhibitors or knockout of *AhR* could effectively rescue BPA-induced metabolic disorders in PCOS mice.

**Conclusions:**

Our results revealed that BPA suppressed GLUT4 expression and induced abnormal glucose metabolism by activating AhR, causing insulin resistance, and is thus a potential contributor to the development of PCOS. Therefore, AhR could be a potential new therapeutic target for PCOS.

Video Abstract

**Supplementary Information:**

The online version contains supplementary material available at 10.1186/s12964-023-01410-y.

## Background

Polycystic ovary syndrome (PCOS) is a common public health important disease in women of reproductive age and is a high-risk factor associated with type 2 diabetes, pregnancy-induced hypertension syndrome, cerebrovascular diseases, and endometrial adenocarcinoma [1–3]. The primary characteristic features of PCOS are ovarian dysfunction, androgen excess, and metabolic disorders including insulin resistance (IR), obesity, dyslipidemia, and abnormal glucose tolerance [4–6].

Studies have shown that nearly 50–60% of PCOS patients have IR, which plays a pivotal role in the occurrence and development of PCOS [7, 8]. Abnormal insulin signal transduction pathways, especially glucose transport defects, could impair glucose disposal, induce a hyperglycemic state, and enhance hyperandrogenemia and inflammatory response [9]. Moreover, IR can interact with hyperandrogenism to form a vicious circulation and, accordingly, develop PCOS symptoms [10].

More importantly, insulin resistance has traditionally been demonstrated as a disorder in whole-body glucose metabolism. It has been reported that the existing obstacle to follicle maturation in PCOS patients is related to abnormal glucose metabolism in granulosa cells [11]. As an important insulin-responsive glucose transporter, GLUT4 plays a critical role in glucose uptake, owing to its high affinity for glucose in various tissues [12]. Additionally, GLUT4 is a major contributor to glucose uptake because of its high affinity for glucose in ovarian granular cells [13, 14].

It has been proposed that IR in PCOS is a consequence of an interplay between genetic predispositions and environmental factors [15]. Lifestyle factors are modifiable and, thus, should be the focus in the treatment and prevention of IR [16]. However, as a key part of packing materials, bisphenol A (BPA) products are widely used in our daily lives. It has been recognized as an endocrine disruptor and is suspected to play a role as a reproductive toxicant [17]. Previous studies have found that serum BPA may be positively associated with PCOS patients [18, 19]. As indicated by evidence of interference with ovarian morphology and ovarian functions, such as steroidogenesis, and particularly folliculogenesis [20, 21]. Whether BPA is a contributor or a consequence of PCOS is unclear. Furthermore, whether BPA exposure triggers IR in PCOS remains unclear. Understanding the relationship between BPA and IR is pivotal for the design and precise regulation of novel therapeutics against PCOS.

Growing evidence indicates that BPA action is initiated by activating specific receptors, inducing transcription factors PPARγ, Nrf2, and aryl hydrocarbon receptor (AhR), thereby directly regulating gene expression [22–25]. The AhR is a ligand-activated transcription factor that plays an important role in several biological processes, including reproduction, immunity, and homoeostasis. Environmental chemicals, such as 2,3,7,8-tetrachlorodibenzo-p-dioxin (TCDD), can bind and activate AhR to regulate transcriptional programmes [26, 27]. These findings imply that BPA may exert important biological effects by binding to AhR, which regulates various metabolic pathways. In support of this notion, AhR-deficient (*AhR*^*−/−*^) mice exhibit undamaged glucose tolerance or insulin responsiveness, unlike wild-type C57BL/6 mice. In the present study, we provide evidence that BPA functions as a ligand for AhR and mediates its metabolic efficacy via glucose transport.

## Materials and methods

### Participants and human samples

Between March 2021 and December 2021, a total of 256 patients aged 18 ~ 40 years at the Reproduction Center, Peking University Third Hospital were enrolled in this study. 136 patients were diagnosed with PCOS according to the 2003 Rotterdam criteria [28]. The other 120 patients were selected from women who visit the center for the vitro fertilization/intracytoplasmic sperm injection (IVF /ICSI), and the cause of marital infertility was male azoospermia. Exclusion criteria include the following: 1) chronic plevic inflammatiory disease; 2) tubal obstruction; 3) endometriosis; 4) had received oral steroids or antidiabetic or diformin; 5) were diagnosed with diabetes, hypertension, chronic renal failure. This study was approved by the Ethics committee of the Peking University Third Hospital(M2021366), and written informed consent was obtained from the enrolled patients.

Peripheral blood samples were collected from all subjects during days 2 ~ 4 of spontaneous cycles after an overnight fast. Levels of serum FSH, luteinizing hormone and SHBG were tested by radioimmunoassays. The levels of estradiol, testosterone, androstenedione and DHEA sulfate were measured using liquid chromatography mass spectrometry (Sciex Triple Quad 6500^+^). The free androgen index was defined as (testosterone (nmol/L) 100)/SHBG (nmol/L). The levels of fasting serum glucose, serum insulin, triglycerides, total cholesterol, high-density lipoprotein cholesterol and low-density lipoprotein cholesterol were measured using an autoanalyzer (Beckman Coulter AU5800). The insulin resistance index (HOMA-IR) was calculated using homeostasis model assessment methods, defined as fasting insulin (mIU/L) × fasting glucose (mmol /L)/22.5.

### Cell lines

A steroidogenic human granulosa cell-like tumor cell line (KGN) was kindly donated by Dr. Yiming Mu (General Hospital of the People s Liberation Army, Beijing, China). The KGN cells were maintained in DMEM/F12 supplemented with 10% FBS, 100 U/ml penicillin and 100 μg/ml streptomycin in a humidified atmosphere of 5% CO_2_ at 37 °C.

The primary ovarian granular cells collected from in IVF/ISCI nonPCOS patients. Follicular fluid were collected, washed in PBS, digested with hyaluronidase, then red blood cell lysis buffer (eBioscience, CA, USA) was added and incubated with the samples for 5 minutes at room temperature. Cells were washed thrice with PBS and then transferred to DMEM/F12 medium (Gibco, USA) supplemented with 10% FBS and incubated at 37 °C for 1 hour. Afterwards, discard of supernatant, addition of complete medium for experiments.

### Reagents

BPA, glucose and kynurenine were purchased from Sigma-Aldrich (MO, USA). DHEA, corn oil, CH223191 were purchased from MedChemExpress (NJ, USA). Insulin and 2-NBDG were purchased from Thermo Fisher (MA, USA). siRNAs targeting human *AhR* and negative control siRNAs (NC) were purchased from RiboBio and transfected into KGN cells using Lipofectamine 2000 Transfection Reagent (Invitrogen).

### Real-time PCR

Total RNA extraction was prepared with TRIzol reagent (15596026, Invitrogen) and reverse transcribed into cDNA by using TransScript First-Strand cDNA Synthesis Supermix (TransGen Biotech Co, Beijing, China). The expression of mRNA for genes of interest were normalized to the β-Actin mRNA level. Values are reported as the mean ± SEM of three independent experiments performed in duplicate. The differences in all parameters were considered statistically significant at a value of *P* < 0.05. The primer sequences were as follows: GLUT4 Forward 5′-GGGCTGAGACAGGGACCATA AC-3′; Reverse 5′-CATGAGCAATGGCATCC CAGAA-3′; CYP1A1 Forward 5′- GATTGGGCACATGCTGACC-3′; Reverse 5′- CTGTCAAGGATGAGCCAGC A-3′; CYP1B1 Forward 5′-TTTCGGCTGCCGCTACA-3′; Reverse 5′- ACTCTTCG TTGTGGCTGAGCA-3′; β-Actin Forward 5′-CCTGAAGTACCCCATCGAGC-3′; Reverse 5′-AAGGAAGGCTGGAAGAGTGC-3′.

### Western blot analysis

Cells were washed with ice-cold PBS and lysed with lysis buffer supplemented with a protease and phosphatase inhibitor cocktail (Thermo Fisher, MA, USA). Protein concentrations were determined using the BCA Protein Assay Kit (Thermo Fisher, MA, USA). Samples of protein and pre-stained molecular weight marker were separated by SDS-PAGE and and transferred to polyvinylidene fluoride (PVDF) membranes (Invitrogen, CA, USA). Individual protein was detected with specific antibodies (1:1000 dilution) and visualized by infrared fluorescent secondary antibodies. The protein bands were visualized and analyzed on Tanon imager (SHH, CHN). Three independent experiments were performed for each analysis.

### Animal studies

Female wild-type C57BL/6 J mice with 3-weeks old were purchased from the Department of Experimental Animal Sciences of the Peking University Health Science Center (Beijing, China). AhR-deficient (*AhR*^*−/−*^) mice were presented by Bo Huang’s laboratory (Institute of Basic Medical Sciences, Chinese Academy of Medical Sciences). For studies, all animal were kept under SPF conditions at the Animal Care and Use Committee of Peking University Health Science Center.

#### BPA-induced toxic model

Three-weeks old female C57BL/6 J mice were equally and randomly divided into 4 groups: the three dose gradients of BPA (5 μg/kg/day, 50 μg/kg/day, 500 μg/kg/day) were intragastric injected respectively for 21 consecutive days. The control group was gavaged daily with same dose of corn oil. Then, part of mice in each group were sacrificed and serum samples were collected for the detection of hormone concentration. The others mice allowed to mate for 1 week (3 dams:1 sire) for checking the number of pups, The females with positive vaginal plugs present the next morning were considered to be on day 0.5 of pregnancy.

### PCOS model

To induce PCOS, 3-week old female C57BL/6 J mice were randomly divided into four groups: control, (intragastric injection, corn oil 0.2 ml /day) BPA (intragastric injection, 50 μg/kg/day); DHEA (subcutaneously injection, 60 mg/kg /day); BPA + DHEA (same injection regimens as those used for monotherapy). All the animals were treated for 21 consecutive days.

#### AhR-deficient model

3-week old female *AhR*^*−/−*^ were equally and randomly divided into 4 groups: the dose gradients of BPA (5 μg/kg/day; 50 μg/kg/day; 500 μg/kg/day) intragastric injection treated for 21 consecutive days. The control group was gavaged daily with same dose of corn oil. Then, the animals from each group were subjected to GTT and ITT.

### Body composition

Body composition analysis was performed using the EchoMRI (EchoMRI 900, Houston, TX, USA). MRI measurements were performed in conscious mice placed in a clear plastic holder without anesthesia or sedation and with a cylindrical plastic insert added to limit movement of the mice. Mice were briefly submitted to a low-intensity electromagnetic field where fat and lean masswere measured.

### Estrous cycle determination

Vaginal smears were collected daily at same time from the 10th day after the treatment. The changes of vaginal epithelial cells were evaluated under a light microscope to determine the estrous cycle. Based on cell type predominance, normal estrus was assigned to 1 of the 4 possible stages of the murine estrous cycle; diestrous (G), proestrus (D), estrus (E) and metestrous(F).

### Ovarian morphology

After the collection of blood samples, the ovarian tissues were harvested and fixed in 10% formalin solution. Tissues were embedded in paraffin and subsequently cut into ultrathin slices (5 μm) using a microtome and stained with hematoxylin and eosin (H&E). The morphology of the ovarian tissues were scanned with Microscopes and Imaging Systems (Leica, GRE).

### Flow cytometry

For flow cytometric analysis, KGN cells or primary ovarian granular cells were seeded and treated with the indicated drugs for 24 h, detached from the plates with Trypsin Solution without EDTA and washed with cold PBS. Glucose uptake was measured using the fluorescent glucose analogue (2-NBDG). Cells were washed with pre-cooled PBS and incubated with 2-NBDG for 20 minutes at 37 °C, protected from light. Cells were washed and collected in PBS and immediately analyzed by flow cytometry, using 488 nm excitation for the 2-NBDG.

### ChIP

KGN cells were plated at a density of 2 × 10^5^ cells and cultured for 24 hours with each treatment condition. After collection of cells, ChIP-qPCR was performed by using ChIP-IT Express Chromatin Immunoprecipitation Kits (Active Motif, CA, USA) according to the manufacturer’s protocol. The antibodies used included anti-AhR (Abcam, Ab2769) and an IgG isotype control (Cell Signaling Technology, 3900). The *Glut4* primer sequences were 5′-ATGACCTCTGGTCACCAAACTG-3′ (sense) and 5′-GATCAGTCAGAAGCATAGGTGAC-3′ (antisense). Real-time PCR amplification was carried out and amplification of target gene is shown as fold enrichment compared to that of irrelevant antibody controls. The results were from 3 independent experiments followed by normalization to input signals and showed as mean ± SEM.

### Immunofluorescence

Cells slides fixed with 4% paraformaldehyde for 20 min at room temperature, blocked in 1% BSA for 30 min. Cells slides were incubated with anti-AhR primary antibodies (Abcam, UK) overnight at 4 °C followed by secondary antibodies Alexa 594-conjugated donkey anti-rabbit IgGs (Invitrogen, CA, USA). Nuclei were stained in DAPI slides were mounted in Fluoromount G (Solarbio, Beijing, China) and stored at 4 °C in the dark. The confocal images were observed under confocal microscope (Nikon, Melville, NY).

### Molecular docking

Molecular docking study was executed applying Schrodinger software package. Firstly, the X-ray crystal structure of AhR was retrieved from Protein Data Bank(PDB code: 1BG1) and prepared with the Protein Preparation Wizard model including the removement of one monomer, the addition of missing hydrogen atoms, the assignment of bond order, assessment of the correct protonation states, and a restrained minimization using the OPLS-2005 force field. After preparing the ligands, molecular docking was carried out using the standard precision (SP) with the default settings. Finally, the pictures were generated using pymol software.

### Statistical analysis

The data shown are from one representative experiment of multiple independent experiments and reported as the mean ± SEM. The statistical significance of differences between two groups was determined by the Student’s t tests or One-way ANOVA followed by Dunnett t-test. *P* values less than 0.05 (*P* < 0.05) were considered significant. Analyses were conducted using GraphPad 7.0 software.

## Results

### Serum BPA levels were increased in patients with PCOS

**Fig. 1:**
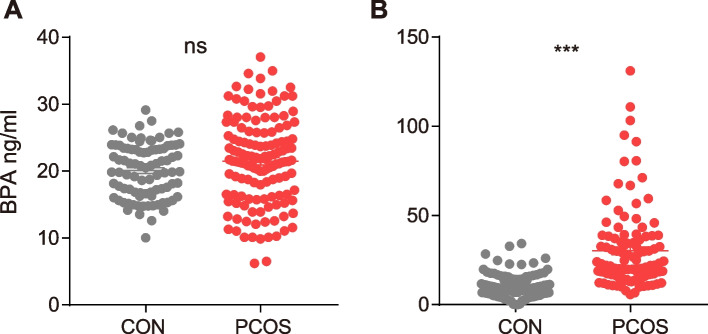
**A** Urine and **B** serum BPA levels between two groups

To determine whether the level of BPA in PCOS is increased, we collected urine and serum samples from PCOS and control groups and recorded demographic features including ages, body mass index (BMI), and hormone levels. The results indicated no notable differences between the groups in terms of age or serum luteinizing hormone, estradiol progesterone, testosterone, and prolactin levels (Table 1). Overall, PCOS patients had higher BMI, serum anti-Müllerian hormone levels, luteinizing hormone / follicle-stimulating hormone ratios and HbAlc than control subjects. In the PCOS group, an increase in BPA levels was observed in urine, but this trend was not significant (Fig. 1 A). This upregulation was significant in serum (Fig. 1 B).

**Table 1:** Demographic data and clinic characteristics between two groups

Variable	Control	PCOS	*P* value	
Age (years)	31.82 ± 0.38	30.84 ± 0.33	0.0516	ns
BMI (kg/m2)	23.40 ± 0.36	25.09 ± 0.42	0.0026	**
AMH (ng/ml)	2.66 ± 0.19	6.00 ± 0.52	< 0.0001	***
LH (IU/L)	7.09 ± 1.63	18.42 ± 9.02	0.2387	ns
FSH (IU/L)	7.84 ± 0.64	6.31 ± 0.17	0.0166	*
LH/FSH ratio	1.03 ± 0.26	2.18 ± 0.14	< 0.0001	***
E2(pg/mL)	178.90 ± 20.60	175.50 ± 10.71	0.8834	ns
P(pg/mL)	2.70 ± 0.91	2.54 ± 0.78	0.8911	ns
T (pg/mL)	1.78 ± 0.45	1.98 ± 0.63	0.7983	ns
PRL (ng/mL)	17.05 ± 2.74	18.02 ± 2.64	0.7999	ns
HbAlc (%)	5.38 ± 0.06	5.52 ± 0.04	0.0476	*

*AMH* anti-Müllerian hormone, *BMI* body mass index, *E2* estradiol, *FSH* Follicle-Stimulating Hormone, *HbAlc* Glycosylated Hemoglobin, *LH* Luteinizing Hormone, *P* progesterone, *PRL* prolactin, *T* testosterone

**Fig. 2:**
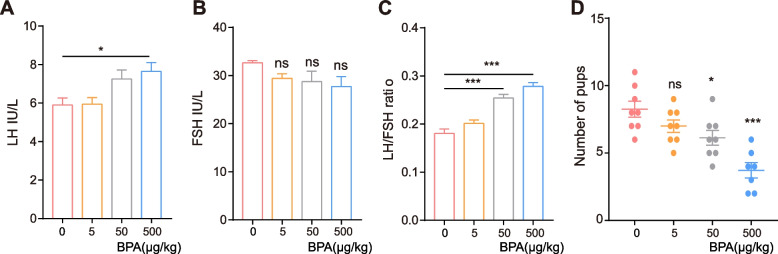
After administration with various concentrations of BPA for 21 days, **A-C** the hormones were detected. **D** The number of pups was monitored. Data are presented as mean ± SEM. * *P* < 0.05 and *** *P* < 0.001

We further explored the correlation between BPA levels and female fertility. Animal experiments were carried out, in which mice were randomly divided into four groups: control, BPA, and low-, middle-, and high-dose groups. As shown in Fig. 2, the changes in LH levels and LH/FSH ratios in the mouse model were higher as the BPA dose increased (Fig. 2 A-C). Next, the female mice were co-housed with male mice, and the number of pups was negatively correlated with BPA dose (Fig. 2 D). Recent studies have also suggested that BPA may influence multiple endocrine-related pathways and induce adverse reproductive outcomes [29, 30]. These results indicate that BPA is a risk factor for PCOS.

### Effects of BPA on body weight, glucose tolerance, and insulin sensitivity in DHEA-induced PCOS mice

**Fig. 3:**
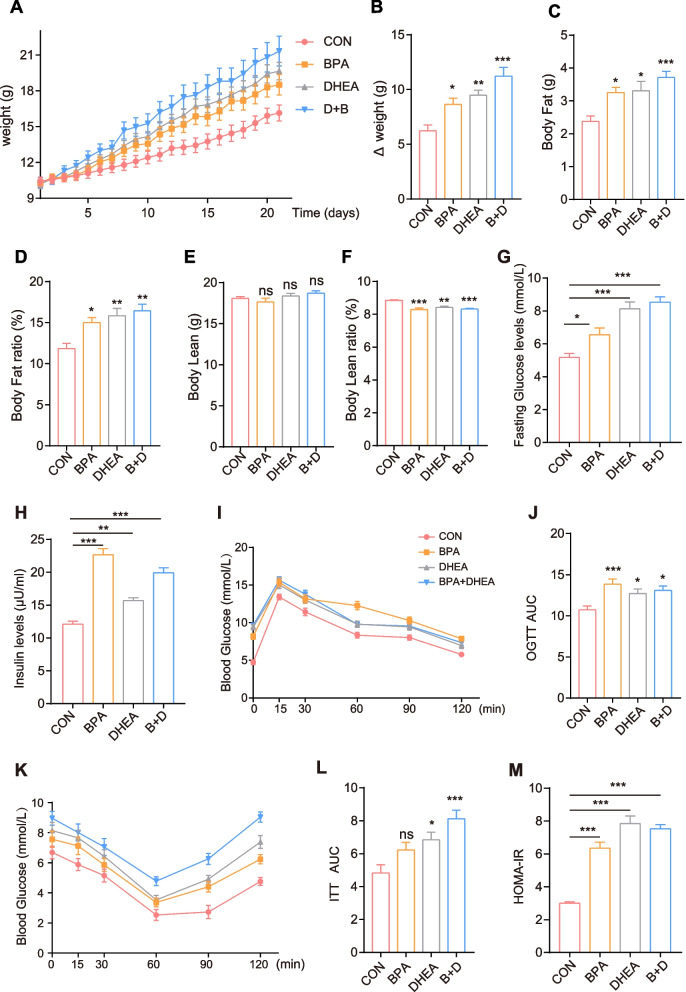
Female mice were randomly divided into four groups: CON (oil), BPA, DHEA, BPA + DHEA (*n* = 10 of each group). During the treatment for 21 days, body weight of mice was measured every day (**A**). **B** Body weight gain, **C** body fat, **D** body fat ratio, **E** body lean and **F** body lean ratio. **G** Blood glucose and **H** insulin levels for indicated treatment. **I**,** J** Oral glucose tolerance tests (OGTTs) (**K, L**) and insulin tolerance tests (ITTs) were performed on mice of the four groups. **M** The homeostasis model assessment of insulin resistance (HOMA-IR) index was calculated of four groups. Data are represented as the mean ± SEM. One-way ANOVA with a Tukey post hoc test. * *P* < 0.05; ** *P* < 0.01, and *** *P* < 0.001

BPA has been recognized as an endocrine-disrupting chemical and is suspected to play a role as a reproductive toxicant [31]. Clinical and preclinical studies have shown that BPA may be positively associated with PCOS [32, 33]. Therefore, in this study, the effects of BPA and/or dehydroepiandrosterone (DHEA) on body weight, insulin sensitivity, and glucose homeostasis were measured in diverse groups. To test the possible role of BPA, we treated mice with control, BPA, DHEA, or BPA + DHEA. We found that the BPA + DHEA mice gained significantly more weight than the DHEA group (Fig. 3 A, B). The highest body fat and body fat ratio were observed in the BPA + DHEA group, as measured by magnetic resonance imaging (Fig. 3 D). Consistent with these results, the body lean ratio decreased in the BPA + DHEA group (Fig. 3 F). Interestingly, fasting blood glucose and fasting serum insulin levels increased in the combined treatment group. Moreover, BPA significantly increased DHEA-induced metabolic disorders. Oral glucose tolerance test (OGTT) or insulin tolerance tests (ITT) revealed delayed glucose clearance and increased area under the curve, indicating that BPA treatment decreased glucose excretion capability. This impairment in glucose tolerance worsened after DHEA administration (Fig. 3 I-L). Homeostatic model assessment of IR (HOMA-IR) levels was significantly higher in the BPA and DHEA groups than in the BPA monotherapy group (Fig. 3 M). Together, these data suggest that the metabolic disturbance effect of BPA exacerbates the impaired glucose tolerance induced by DHEA treatment in mice.

### BPA treatment disrupted the estrous cycle and ovarian function

**Fig. 4:**
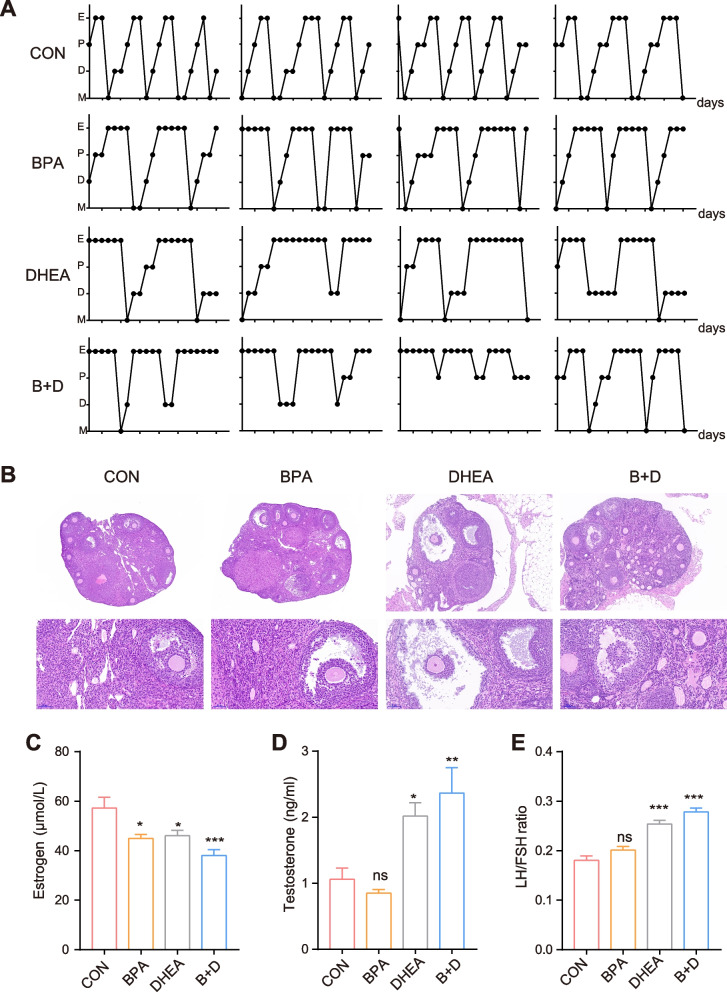
**A** Representative estrous cycles were determined after another 10 days. E, estrus; P, proestrus; D, diestrus; M, metestrus. **B** Representative ovary sections were stained with H&E (scale bar = 500 μm). The serum levels of **C** estrogen, **D** testosterone, and **E** LH/FSH ratio. Data are presented as mean ± SEM. *P* values were calculated using one-way ANOVA, * *P* < 0.05; *** *P* < 0.001

Next, we studied the pathophysiological changes underlying these BPA-mediated metabolic disorders. Estrous cycles were measured to determine the effects of BPA and/or DHEA on ovarian function. Mice in the control group exhibited regular estrous cycles of 4–5 days, whereas most mice in the BPA and/or DHEA groups were in the diestrus phase (Fig. 4 A). Consistently, hematoxylin-eosin staining was performed to determine alterations in ovarian pathology in the diverse groups. No structural abnormalities or ovarian cysts were observed in the control group (Fig. 4 B). Similar to DHEA alone, BPA treatment resulted in a higher number of cystic follicles, thinner granulosa cell layers, and a lower number of corpora lutea compared to the control treatment. At the same time, BPA intensified the DHEA-induced ovarian abnormalities in mice. Furthermore, serum hormone levels were measured. In the mice treated with BPA in the presence or absence of DHEA, BPA significantly decreased serum eE2 levels, but did not have an obvious effect on testosterone levels (Fig. 4 C-E). Taken together, these results suggest that BPA exacerbates ovarian dysfunction and pathological damage to ovarian tissues in PCOS mice.

### AhR was involved in BPA-induced insulin resistance in ovarian granular cells

**Fig. 5:**
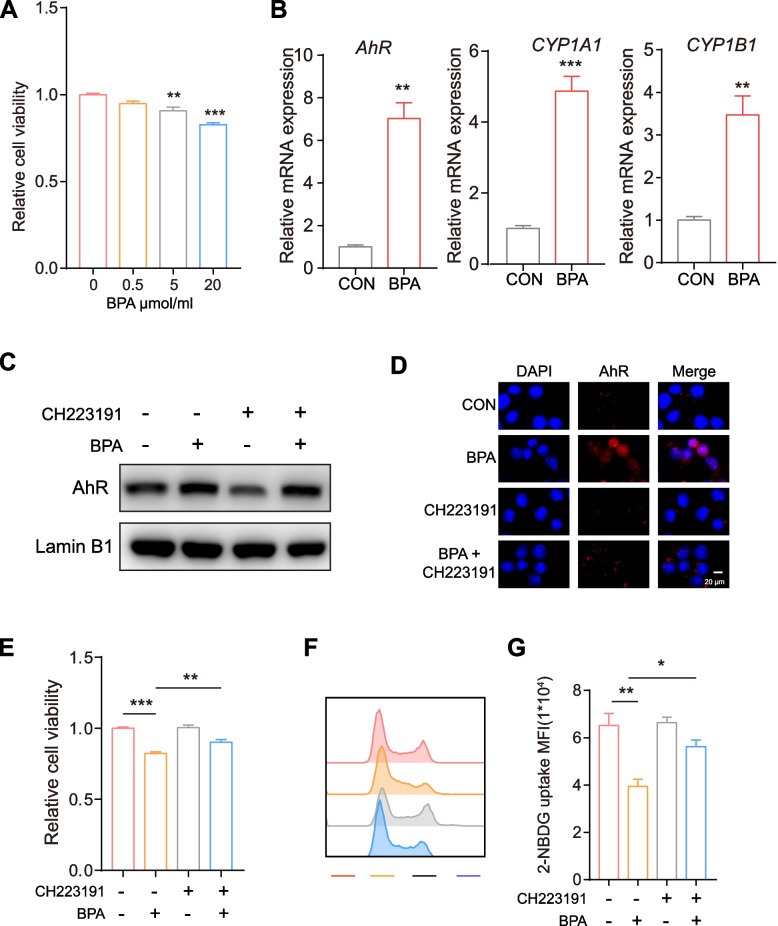
**A** Cell vitality was observed with CCK8. **B** Elevated AhR, Cyp1a1 and Cyp1b1 expression were confirmed by qPCR. KGN cells were treated with PBS, BPA (10 μmol/L), CH223191(10 μmol/L) or BPA/CH223191 for 24 h, and the resulting effects on AhR location were determined by (**C**) western blots and (**D**) immunofluorescence. **E** Cell vitality and (**F**) glucose uptake was analysed. Values are presented as mean ± SEM; *p < 0.05; **p < 0.01; ***p < 0.001

Insulin resistance is one of the most significant factors that cause ovulation disorders in PCOS patients, and is mainly ascribed to proliferation inhibition and dysfunction of ovarian granular cells and subsequent inhibition of normal growth and ovulation of follicles [34]. BPA treatment reduced the viability of KGN and primary ovarian granulosa cells (Fig. 5 A).

The aryl hydrocarbon receptor (AhR) is a ligand-activated transcription factor that can be activated by exogenous xenobiotics and metabolic cues to control transcriptional programs, including the insulin-signaling pathway, in a wide variety of cells [35, 36]. To better understand the molecular mechanism, we investigated whether BPA alters the expression of *AhR* in ovarian granulosa cells. Genome-wide transcriptional profiling and qPCR analysis revealed a potential AhR response in ovarian granular cells exposed to BPA (Fig. 5 B, C). We also found that the addition of BPA to ovarian granulosa cells resulted in AhR translocation to the nucleus, as demonstrated by western blotting and fluorescence microscopy (Fig. 5 D, E). As nuclear localization reflects an active form of AhR, this result suggests that AhR signaling might be activated during BPA incubation. Furthermore, combining BPA with the AhR inhibitor CH223191 inhibited the translocation of AhR to the nucleus, as confirmed by cell viability and glucose uptake assays (Fig. 5 F, G).

### BPA-mediated activation of AhR induced abnormal transcription of GLUT4 in ovarian granular cells

**Fig. 6:**
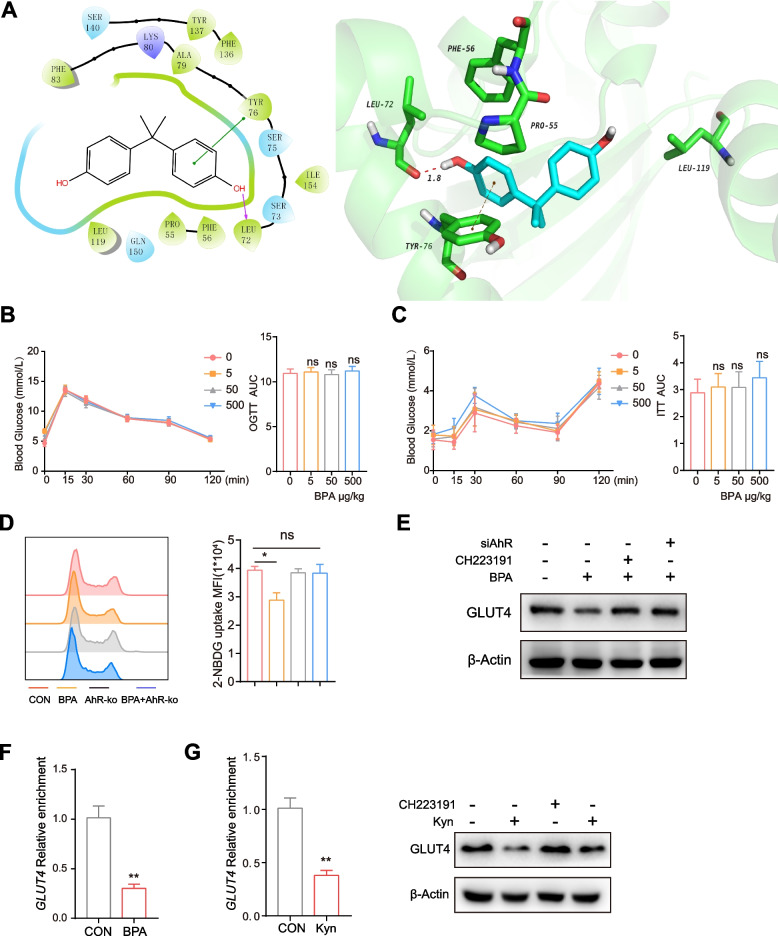
**A** 2D and 3D interactions between BPA and AhR. After administration with various concentrations of BPA for 21 days, **B** Oral glucose tolerance tests (OGTTs) (**C**) and insulin tolerance tests (ITTs) were performed on mice of the four groups. **D** Glucose uptake was evaluated by measuring 2-NBDG uptake using flow cytometry (FACS). **E** Western blots showing the expression of GLUT4 after the indicated treatment. **F** ChIP-qPCR analysis was performed with an anti-AhR antibody and GLUT4 promotor-specific primers. Data are presented as values relative to the control group value. **G** mRNA expression was determined by qPCR. Protein expression was determined by western blotting. The data shown are representative of three independent experiments, and the error bars represent the mean ± SEM. *P < 0.05; **P < 0.01; ***P < 0.001; ns, not significant

To elucidate the binding mode of BPA with the AhR PAS-A domain, a docking study was carried out on the basis of the crystal structure of the AhR homo dimer (PDB code:1BG1). These interactions confirmed the activity of BPA toward AhR protein (Fig. 6 A). Next, we assessed the role of AhR in BPA-mediated insulin resistance. In contrast to those of wild-type C57BL/6 mice, the OGTT and ITT of *AhR*^*−/−*^mice were indistinguishable after treatment with different doses of BPA, suggesting that BPA-mediated insulin resistance depends on the activation of AhR (Fig. 6 B, C). Consistent with these results, there was no difference in glucose uptake between *AhR* knockdown KGN cells treated with or without BPA (Fig. 6 D). Among the genes affected by BPA, glucose transporter 4 (GLUT4) was downregulated as a result of BPA treatment in KGN cells (Fig. 6 E).

To further demonstrate the role of AhR in BPA-mediated insulin resistance, the expression of GLUT4 was detected. The chromatin immunoprecipitation (ChIP)-qPCR data showed that BPA facilitated the binding of AhR to the suppressor of *GLUT4* genes, causing a significant decrease in gene expression (Fig. 6 F). To further confirm insulin resistance in AhR-driven ovarian granular cells, we tested the endogenous AhR agonist kynurenine (Kyn). Consistently, Kyn downregulated the mRNA and protein expression of *GLUT4* (Fig. 6 G). Collectively, these data suggest that the activation of AhR-mediated signaling contributes to BPA-induced glucose transport disorder in ovarian granular cells.

## Discussion

Infertility has become the third-most common disease after tumors and cardiovascular and cerebrovascular diseases [37, 38], and its prevalence in women with PCOS ranges from 70 to 80% [39]. Increased infertility is due to many factors such as environment, diet, living habits, and mental stress, and one of the main reasons for this is unsuspected environmental factors [40]. PCOS is the primary cause of anovulatory infertility, with a prevalence of 5.6% in women of reproductive age women in China [41–45]. A higher incidence of PCOS is characterized by abnormal glucose metabolism, insulin resistance, hyperandrogenism, ovarian dysfunction, and obesity, and is associated with a higher risk of pregnancy and birth complications [46, 47]. Women with PCOS with high BMI and IR have an increased risk of spontaneous abortion when receiving assisted reproductive technology [48]. Recent studies suggest that with or without diabetes, the administration of GLP-1 receptor agonists alone or in combination with metformin generally improves weight and glucose parameters in women with obesity and PCOS [49]. Changes in intermediate metabolic pathway substances in the follicular fluid of PCOS patients and the impairment of mitochondrial function may be involved in the regulation of reproductive and metabolic abnormalities in PCOS patients through epigenetic and protein post-translational modifications [50–52].

As the most common endocrine-disrupting chemical, BPA was the first synthetic estrogen with a non-steroid hormone structure [53, 54]. Emerging evidence has demonstrated that BPA causes significant changes in normal metabolism [55–57]. Research demonstrates that BPA can block the tricarboxylic acid cycle, and BPA also regulates the nuclear receptor LXR, causing hypoglycemia and consequently affecting the normal metabolic functions of the liver [58]. Other studies have shown that BPA causes mitochondrial dysfunction, which may cause a compensatory shift from oxidative metabolism to glycolytic metabolism through alterations in the insulin signaling pathway [59]. Several studies have confirmed an association between BPA and PCOS development. Case-control study obtained the same result and suggested that BPA may play a major role in the pathogenesis of PCOS, especially the occurrence of IR [60]. Moreover, research indicates that metformin has additional benefits against BPA-induced metabolism imbalance [61], but the exact mechanism is unknown. Several animal studies have examined the effects of prenatal exposure to BPA and have reported changes such as an increased incidence of ovarian cysts, delayed first ovulation, and reduced corpus luteum numbers [62]. Moreover, a common finding in animals exposed to BPA in early developmental stages is the alteration in ovarian morphology, similar to the appearance of polycystic ovaries [63, 64]. Taken together these results suggest that BPA levels are higher in women with PCOS, while it cannot be ruled out that BPA and PCOS have a causal bidirectional effect.

Recent studies have linked BPA exposure to insulin resistance and glucose intolerance, and BPA significantly decreased the protein levels of insulin signaling molecules such as IR, IRS, AKT, AS160, and GLUT4, thus decreasing glucose uptake and oxidation in the muscle [65, 66]. In addition, another study in a mouse pregnancy model indicated that the upregulation of GLUT1 expression is closely related to the effect of BPA; in this study, the mice were treated with BPA during the 20 days of pregnancy and for 1 month of the pregestational period [67]. In our study, we found that BPA blocks GLUT4 expression in granulosa cells, and the oxidative energy supply of glucose is defective, which may trigger the activation of alternative metabolic pathways, such as the lipid metabolic pathway, to compensate for energy demand. The changes in glycolipid metabolites in follicular fluid affect follicular development and ovum quality. This may be the reason why exposure to BPA decreases fertility.

Limitations of this study are PCOS is a highly heterogeneous disease and possible pathogenic factors are diverse. There are several potential endocrine targets for diagnosis and treatment of PCOS. However, the role and influence of these endocrine indicators include GILU4 and AhR on PCOS classification and diagnosis need to be further clarified in the future investigation. In the meantime, the role of BPA-affected PCOS on various links of reproduction can be elucidated from multiple dimensions such as ovulation disorder, endometrial receptivity, metabolic disorder and IVF outcome.

**Fig. 7:**
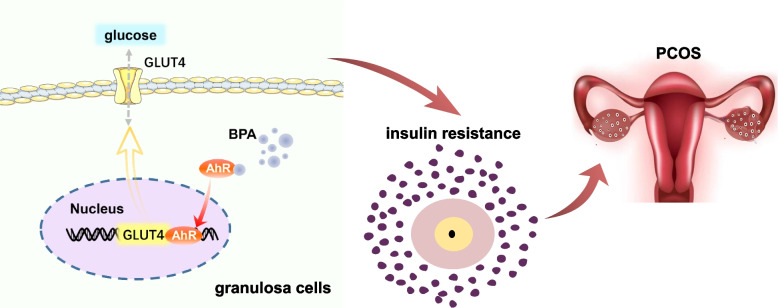
Schematic diagram. Schematic model indicating at which the BPA will disturb ovarian granular cells glycometabolism exacerbating PCOS. BPA suppressed GLUT4 expression and induced abnormal glucose metabolism by activating AhR, causing insulin resistance, and is thus a potential contributor to the development of PCOS

The environmental sensor AhR has been discovered as a high-affinity dioxin and other heterogeneous organism binding protein and is characterized as a multifunctional ligand-activated transcription factor. Several studies have demonstrated that AhR acts as a potential regulator of glucose homeostasis metabolism [35, 68, 69]. In addition, our data suggest that exposure to BPA may bind to AhR, enable its activity, and alter glucose transport capacity. Genetic depletion of AhR diminished glucose transport, OGTT, and ITT responses to BPA-induced changes. This study provided evidence that AhR reduces GLUT4 expression, thereby impairing glucose transport. Intrinsically, the reduction of GLUT4 expression could potentially increase insulin resistance [70]. GLUT4 expression is decreased in the endometrium of PCOS patients, resulting in abnormal glucose utilization in their endometrium [71]. In this case, BPA further activates AhR and decreases the expression of GLUT4, which may trigger IR, thus creating a PCOS cascade. Thus, targeting AhR provides a strategy for the treatment of PCOS (Fig. 7). It is essential that more longitudinal studies and basic experimental research be conducted with increased sample sizes and in a variety of human populations to establish a strong link between BPA exposure and human health outcomes.

## Conclusions

In this study, we demonstrated a novel role for BPA in exacerbating abnormal glucose metabolic disorders in PCOS. Abnormal fertility and increased LH/FSH ratio were observed in female mice exposed to BPA. Further studies showed that BPA aggravated the changes in body weight and body fat, increased blood sugar, impaired glucose tolerance, estrous cycle disorders, sexual hormone disturbance, and ovarian polycystic lesions in DHEA-induced PCOS model rats. The significant finding of this study is that AhR is primarily responsible for inducing IR in ovarian granular cells. This effect was verified by the fact that *AhR*^*−/−*^ mice induced under the same conditions did not develop PCOS-related lesions.

### Supplementary Information

**Additional file 1.**

## Data Availability

The data that support the findings of this study are available from the corresponding author upon reasonable request.

## Abbreviations

AhR: aryl hydrocarbon receptor
AMH: anti-Müllerian hormone
BMI: body mass index
BPA: bisphenol A
DHEA: dehydroepiandrosterone
E2: estradiol
FSH: follicle-stimulating hormone
HbAlc: Glycosylated Hemoglobin
IR: insulin resistance
ITT: insulin tolerance tests
LH: luteinizing hormone
OGTT: oral glucose tolerance test
P: progesterone
PCOS: polycystic ovary syndrome
PRL: prolactin
T: testosterone
