# Supplementary figures and images for: Bisphenol a downregulates GLUT4 expression by activating aryl hydrocarbon receptor to exacerbate polycystic ovary syndrome
Source: Cell Commun Signal. 2024 Jan 10;22:28. doi: 10.1186/s12964-023-01410-y (PMC10782693; doi:10.1186/s12964-023-01410-y)

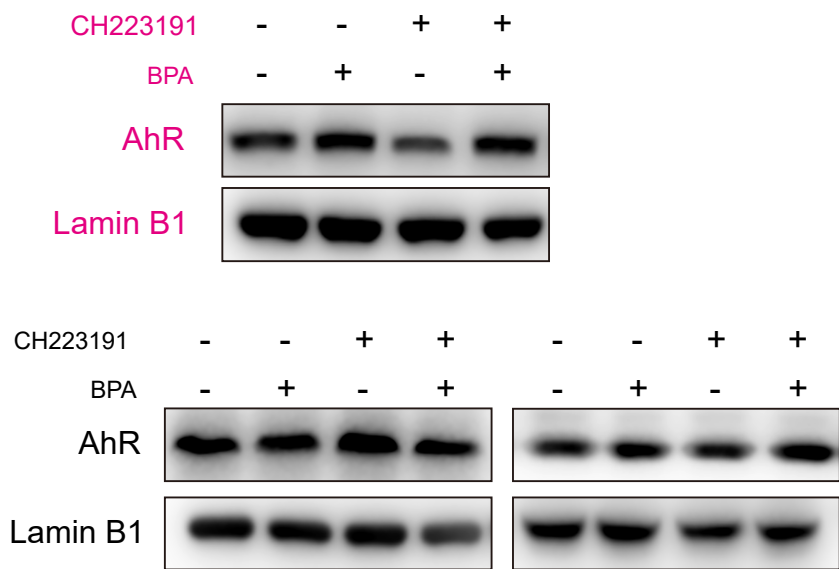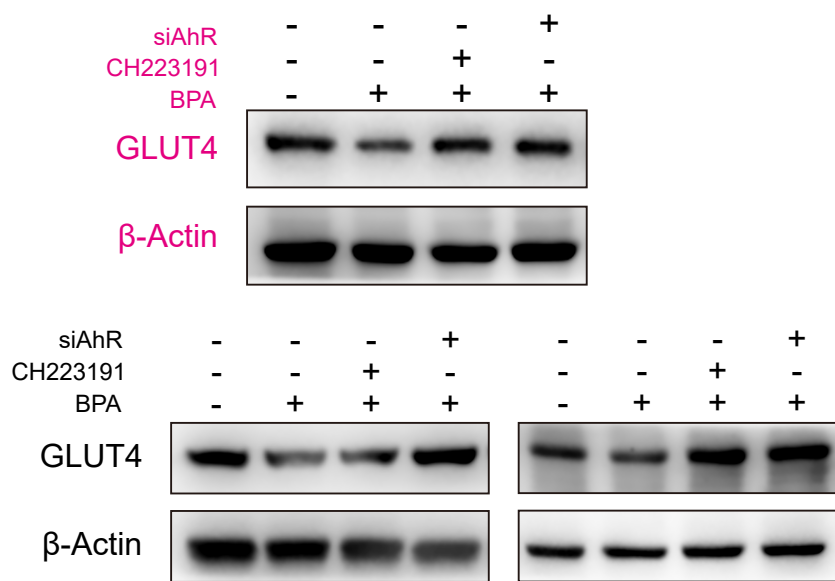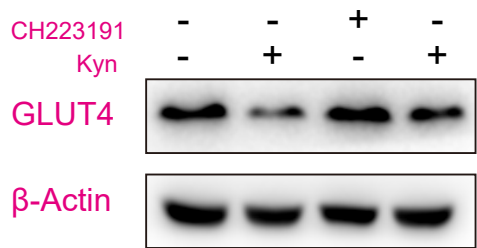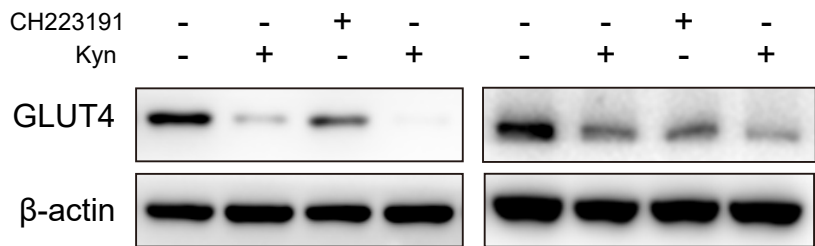

Supplement: Supplementary file 2 — Additional file 1. [file 12964_2023_1410_MOESM1_ESM.pdf]
